# Improving the quality of sexual history disclosure on sex offenders: Emphasis on a polygraph examination

**DOI:** 10.1371/journal.pone.0239046

**Published:** 2020-09-17

**Authors:** Sue Hyun Jung, Min Jin Jin, Jang-Kyu Lee, Hee-Song Kim, Hyung-Ki Ji, Ki-Pyoung Kim, Myoung-Ho Hyun, Hyeon-Gi Hong

**Affiliations:** 1 Division of Forensic Psychology, National Forensic Service, Wonju-si, Gangwon-do, Republic of Korea; 2 Institute of General Education, Kongju National University, Gongju, Republic of Korea; 3 Institute of Forensic Psychiatry Ministry of Justice, Gongju-si, Chungcheongnam-do, Republic of Korea; Sapienza, University of Rome, ITALY

## Abstract

The increasing recidivism rate of sex offenders indicates potential problems in existing recidivism programs. The present study was conducted to determine whether the polygraph examination is a useful technique to obtain a sex offender’s concealed past sexual history. We collected fifty-two sex offenders’ data and analyzed it. Among the 52 participants, the court ordered 26 sex offenders to take the psychiatric evaluation and the polygraph test. The other half were prisoners at the hospital who were currently undergoing treatment. The participants in the polygraph group disclosed more deviant sexual behaviors and paraphilia interests/behaviors than the comparison group. Thus, the polygraph examination is a powerful tool that can encourage sex offenders to disclose hidden information to help create suitable psychological therapy programs for preventing recidivism in the future.

## Introduction

Sexual violence has been a problem in our society, and the public’s concern in sexual abuse has increased. The incidence of sexual violence continues to rise, and the number of offenders who re-entered to prison after committing sex offenses is expanding in South Korea. According to a criminal analysis on the Korea Supreme Public Prosecutor's Office [1], the number of sexual crimes has gone up by 94.7 percent over the past decade, from 16,129 in 2008 to 32,824 in 2017. Violent crimes, including murder, robbery, and arson, have declined over the past decade, while sexual crimes have increased by 1.9 times [1]. The recidivism rate has more than doubled from 3 percent in 2011 to 7 percent in 2014 [1]. In other countries, the situation was slightly different from South Korea. Hanson and Bussiere [2] reviewed 61 recidivism studies and found that only 13.4% of sex offenders committed new crimes within three to four years of their release. However, the actual number of crimes did not reflect on the crime rate because of unreported crimes. According to 20-year follow-up studies [3, 4], the actual rate of sex offender recidivism might increase to 30 to 40%.

Over the past several years, many policies, such as community notifications, GPS tracking, and medication use to reduce sexual arousal, have been implemented to reduce and prevent sex offender recidivism. However, these systems may not be eliminating the offender’s fundamental motivation to commit the offense [5, 6], even though it may reduce crimes by controlling and monitoring their behaviors.

Although current prevention systems are competent in managing offenders’ external behaviors, it is questionable whether these programs are beneficial to offenders. One way to resolve the limitation would be sex offender treatments. Sex offender treatment helps offenders confront deviant sexual interest, cognitive distortion, and sexual knowledge; therefore, it is vital to provide proper treatment to offenders. To do this, it is necessary to understand the subject’s past sexual behavior and habits. In most cases, however, collecting information still relies on a self-report questionnaire, which is profoundly affected by participants’ honesty [7]. Offenders may be deceitfully responding to specific questions. Namely, honesty is regarded as an essential precondition for the successful collection of offenders’ information. Grubin [8] argued that the full disclosure of sexual history offers an opportunity to understand an offender’s triggers, tendencies, and sexual interest. Therefore, it is necessary to get reliable and credible information from sex offenders to develop sex offender treatment.

According to the study on Abel, Becker, Cunningham-Rathner, Mittelman, & Rouleau [9], sex offenders tend to substantially under-report on sexually deviant thoughts and behaviors. Furthermore, self-reports are influenced by response bias, gender, educational background, ethnicity, etc [10]. Therefore, an additional measurement tool is needed to allow sex offenders to honestly bring out all of their past sexual experience information without hiding it.

The Sexual History Disclosure Examination (SHDE) interview with a polygraph is a part of Post-Conviction Sex Offender Testing (PCSOT). It is an effective method to encourage offenders to disclose the previously unknown information by using a polygraph [11–13].

SHDE interviews with polygraph tests help find the offense-specific and offender-related information through physiological recording [14–18]. The SHDE interview with polygraph is a useful technique for exploring examinee's previous sexual involvement by encouraging disclosure in the knowledge that they will be having a polygraph test. Murray, O'Connell, Schmid, & Perry [19] reported that students showed a more accurate response regarding their smoking experience when utilizing a biological test than a self-report. Thus, obtaining information from the SHDE interview with a polygraph will be more credible and reliable. This will also allow sex offender treatment providers to develop effective treatment [20].

Several studies have conducted to identify the effectiveness of the SHDE interview with polygraph. Buschman, Bogaerts, Foulger, Wilcox, Sosnowski, and Cushman [21] found that participants reported a more significant number of deviant behaviors during polygraph sessions. Another study also showed that sex offender inmates reported more victims and offenses during polygraph sessions [22]. Similarly, Wilcox & Sosnowski [23] found that medium- to high-risk sex offenders disclosed significantly more information during polygraph examinations than participants' pre-sentence file records.

Regardless of the outcomes, these studies have several limitations. First, the participants in these studies were in the middle of the treatments, which might reduce deviant thoughts and behaviors. In other words, the increased number of disclosures might be affected by psychological treatments, not by the polygraph. According to the studies [24, 25], receiving treatment helps the offender drop the level of denial on past offenses. Second, previous studies did not include a comparison group. Therefore, in sum, it is still controversial whether the increased number of disclosures on sexual behaviors among offenders is purely due to the polygraph or other factors. To identify this, we included a comparison group in this study.

National Forensic Psychiatric Hospital center utilized a polygraph test in the SHDE interview to identify offenders’ sexual history since 2015. Before that, they only conducted a self-report to detect sexual history information of offenders. In this study, we used these collected data to discover the effectiveness of SHDE interview with polygraph. Thus, we investigated whether the SHDE interview with a polygraph test encourages sex offenders to disclose more sexual history information. Specifically, we evaluated the polygraph effect by comparing a polygraph group and a self-report group on the SHDE interview. The information obtained in this study was the number of sexual deviances, age at onset of offense, paraphilia interests/behaviors, and victim-related data.

The present study hypothesized that sex offenders who underwent polygraph examination would be more likely to report deviant behavior and interest than sex offenders who only had a self-report. These collected data and results can eventually help improve sex offender treatments and supervision in the future.

## Materials and methods

### Participants

The present study analyzed the records of 52 adult sex offenders. The data was collected from the electronic records of participants. Both groups had the same sexual history disclosure examination interview, which consisted of the questions on examinee’s age, sex, physical or mental health status, and sexual history records.

The study data was collected from the preexisting records of offenders who visited the National Forensic Psychiatric Hospital between September 2015 and January 2017. In the polygraph group, we gathered the registration of 26 sex offenders who were ordered by the court to get a polygraph test. These offenders were sent from the court to the hospital for the psychiatric evaluation to determine their guilt. They stayed at the hospital for a month and received psychological assessments, including sexual history examination interview and the polygraph test. The comparison group data was collected from the inmates imprisoned at that time for the guilty of sexual assaults after conviction. These inmates had to undergo regular treatments and were required to receive psychiatric investigations, including the SHDE interview, to check the effectiveness of treatments.

All participants in the study were charged with sexual assaults and had a history of more than one sex crime against adults. However, in this study, the obtained information was limited, as only the age and offense types were identified. Therefore, other information except for these two findings was not included in the analysis. The in-depth explanation will be provided later in the limitation section.

The mean age of participants was 39.54 years (standard deviation, SD = 12.98 years). All participants were male, and the types of offenses that sex offenders committed were classified into the following three categories: 26 sexual assaults with penetration, which including rape, quasi-rape, and attempted rape (against adults: 26.9%, against children: 73.0%), 25 sexual assaults without penetration (against adults: 32.0%, against children: 68.0%), and one underwear theft.

### Ethics statement

The Institutional Review Board of the National Forensic Service (906-150327-HR-002-01) approved the study. The written consent from the study participants was not obtained because the data records consisted of the de-identified secondary data for research purposes. The requirement for informed consent was waived due to its retrospective nature.

### Sexual history disclosure questionnaire

The Sexual History Disclosure questionnaire is an instrument that helps explore sex offenders' past activities on sexual interest/behaviors. The original questionnaire consists of 42 items that measure the undisclosed sexual acts of sex offenders [26]. Compare to this, we added and modified questions to take into account the cultural background of Korea. During the 1980s, the Korean sex industry proliferated into the entertainment industry and the easy access to purchased sexual services to people [27]. For this reason, the series of questions were added, such as sexual fantasies with celebrities, hiring a prostitute, and watching pornography to the Sexual History Disclosure questionnaire. Therefore, the questionnaire's total number consisted of 54 items, and the participants answered each of every question.

The contents of items were as follows: age, history of sexual life with partner, current and prior sexual offenses, previous non-sexual violence, prior noncontact sex offenses, victim’s age/gender, and offender's age at onset of offense. Participants responded yes or no to each question. They also answered details about their sexual history, such as the victim’s age and gender, the time of the first offense, and the kind of deviant sexual interests. For example, questions such as “Have you ever masturbated in public?" or "Have you ever had sexual intercourse with a child under 13 years of age?" were asked. A list of types of questionnaires is available in the S1 Appendix.

### Computerized polygraph system

The polygraph equipment used in this study was the Lafayette Instrument LX-5000 (Lafayette Instrument Co., North Lafayette, IN, USA), which records the physiological phenomena respiration, heart rate, blood pressure, and skin conductance. The polygraph assesses the examinee's responses while he/she is connected to sensors that transmit data on physiological phenomena.

### Polygraph technique

Comparison Question Technique (CQT) is a frequently used method on polygraph examinations in many countries. The validity and reliability of the technique have been proven in prior studies [28]. The CQT is composed of the three types of questions: relevant, comparison, and neutral questions. The examiner asked several questions to offenders related to his sexual issues while measuring the psychophysiological responses. Deception and non-deception indicated examinee's psychophysiological responses obtained from the relevant and comparison questions [29]. Fundamentally, the examiner compared the examinee's physiological responses elicited by relevant and comparison questions.

### Procedure

The data of the sexual history disclosure examination interview was collected from the National Forensic Hospital, and the data on polygraph test was gathered by the National Forensic Service. The National Forensic Service's IRB approved the retrospective chart review. The data collection procedure was carried out as follows.

First, the participants in the polygraph group were provided with detailed instructions regarding the process. They were informed that offenders' information would be protected and not used for evidence in the trial process. Afterward, the participants completed the sexual history disclosure questionnaires. All participants were encouraged to answer the questions honestly. After completing the questionnaires, the clinical psychologist notified that a polygraph examination would be conducted to determine whether responses would be correct or not. Before performing the polygraph test, the clinical psychologist suggested it would be the last chance to disclose any information they had not reported earlier. Then, participants and clinical psychologist reviewed the sexual history questionnaires together. Next, a polygraph test was conducted.

Before the start of the test, the examiner explained the polygraph records physiological responses in order to determine the accuracy of the examinee’s answers to the test questions. The polygraph examiner selected three questions to ask and started the test. An example of the question was provided as: "Have you ever forced someone under the age of 15 to have sexual intercourse?” The examinees were asked three questions on each issue.

While the polygraph group was undergoing the polygraph testing, the self-report only group completed the sexual history questionnaire and confirmed his/her answers during the review discussion. They were also encouraged to disclose any new sexual history that was not previously reported. If they did not want to make any changes, the interview was ended.

### Data analysis

In this study, we categorized the data into four parts: Previous deviant sexual behavior, Paraphilia interest/behavior, Age at the early onset of criminal behavior, and Victim’s age. Previous deviant sexual behavior consisted of contents about aberrant sexual behaviors that participants have committed before. The paraphilia interest/behavior included a list of paraphilic interests in DSM-5. The participants were asked two questions: “How old were you when you did this?” (Age at the early onset of criminal behavior), and “How old was the victim?” (Victim’s age). The age at the early onset of criminal behavior means the offenders’ age when he first committed offenses. Victim’s age is defined as the average victim’s age, which offenders convicted of crimes in the past. These two questions were included in all questions. SPSS 22.0 was used to analyze the data in this study. The demographic information of participants was analyzed using descriptive statistics. Repeated ANOVA was conducted to verify the main and interaction effects of time and group. The simple main effect was analyzed using a paired *t*-test.

## Results

### Demographic characteristics

The participants were 52 adult sex offenders (26 in the polygraph examination group and 26 in the self-report group). The mean age of the study population was 39.55 years (SD = 12.98 years). The difference in age between the two groups was not significant *F*(1,50) = .211, *p =* .648. No participants had any history of psychiatric disability, drug or alcohol abuse problems.

### Homogeneity test

The means and SD scores of the polygraph and self-report groups are presented in Table 1. The *t*-test was used to determine the homogeneity of pre-scores between the two groups. There were no significant differences on pre-scores; the total number of previous deviant sexual behavior, *t*(50) = -1.366, *p* = .178, paraphilia interest/behavior, *t*(50) = -.278, *p* = .783, age at early onset of criminal behavior, *t*(50) = .336, *p* = .783, and victim's age, *t*(38) = .697, *p =* .*490*.

**Table 1:** Pre- and post- means and standard deviation score of each group.

	Polygraph group (N = 26)		Self-report group (N = 26)	
	M (SD)		M(SD)	
	Pre	Post	Pre	Post
**Number of previous deviant sexual behavior**	7.88 (4.64)	9.96 (5.70)	9.61 (4.49)	9.34 (4.99)
**Paraphilia interest/behavior**	2.12 (2.23)	2.92 (2.96)	2.27 (1.73)	2.08 (2.33)
**Age at early onset of criminal behavior**	21.09 (5.04)	21.10 (5.55)	20.57 (6.29)	20.17 (4.79)
**Victim's age**	23.72 (7.22)	23.23 (6.77)	22.23 (6.29)	23.74 (7.54)

### Sexual history disclosure

**Total number of previous deviant sexual behaviors.** To determine a group difference in the total number of deviant sexual behaviors, repeated-measures ANOVA was conducted. Time (pre, post) and group (polygraph, self-report) were the independent variables, and the score on deviant sexual behaviors was the dependent variable. Results showed that the main effect on time was significant, *F*(1,50) = 9.040, *p <* .005, *η*^*2*^ *=* .153, but not on group, *F*(1,50) = .171, *p <* .681, *η*^*2*^ *=* .003. A group x time interaction effect was also found, *F*(1,50) = 15.228, *p <* .001, *η*^*2*^ *=* .233. These results are described in Table 2.

**Table 2:** Repeated ANOVA on the total number of previous deviant sexual behaviors.

Source of variability	*df*	*SS*	*MS*	*F*	*η²*	*p*
**Between groups**						
**group**	1	8.087	8.087	.171	.003	.681
**error**	50	2362.17	47.243			
**Within groups**						
**Time of the measurement**	1	21.240	21.240	9.040	.153	.004
**time x groups**	1	35.779	35.779	15.228	.233	.000
**error**	50	117.481	2.350			

Significant at the p<0.05 level.

A paired *t*-test was performed for further analysis. The polygraph group showed significant increase in deviant sexual behaviors after notification of polygraph test, *t*(25) = -4.215, *p <* .001, but the self-reported group showed no changes, *t*(25) = .782, *p* = .442. These results suggest that the polygraph group disclosed more sexual deviant behaviors after the clinical psychologist notified the polygraph test. In contrast, the self-reported group did not show any changes between pre and post scores.

### Total number of paraphilia interest/behaviors

There were no significant main effects on time, *F*(1,50) = 2.246, *p* = .141., *η*^*2*^ *=* .043, and group, *F*(1,50) = 3.11, *p* = .579, *η*^*2*^ *=* .006 were found. However, a significant time x group interaction effect on paraphilia interest/behaviors was observed, *F*(1,50) = 5.905, *p* < .05, *η*^*2*^ *=* .106. The results are shown in Table 3.

**Table 3:** Repeated ANOVA on the number of paraphilia interest/behavior.

Source of variability	*df*	*SS*	*MS*	*F*	*η²*	*p*
**Between groups**						
**group**	1	3.115	3.115	.311	.006	.579
**error**	50	500.423	10.008			
**Within groups**						
**the time of measurement**	1	2.462	2.462	2.236	.043	.141
**time x groups**	1	6.500	6.500	5.905	.106	.019
**error**	50	55.038	1.101			

Significant at the p<0.05 level.

To further analyze, a paired *t*-test was conducted. Polygraph group showed a significant increase in the paraphilia interest/behavior scores, *t*(25) = -2.361, *p* < .05, but the self-report group did not show any effects, *t*(25) = .840, *p* = .409. These results mean that the polygraph group disclosed more paraphilia interest/behaviors after being informed of the clinical psychologist's polygraph test.

### Mean scores of offenders’ age at early onset of criminal behavior

There were no significant main effect on time *F*(1,50) = .177, *p* = .676, *η*^*2*^ *=* .004, and group, *F*(1,50) = .260, *p* = .612, *η*^*2*^ *=* .005 were found. Neither significant group x time interaction effect was found on the mean scores of offenders’ age at the early onset of criminal behavior, *F*(1,50) = .204, *p* = .654, *η*^*2*^ *=* .004. The results are also represented on Table 4.

**Table 4:** Repeated measure ANOVA on mean scores of offenders’ age at early onset of criminal behavior.

Source of variability	*df*	*SS*	*MS*	*F*	*η²*	*p*
Between groups						
group	1	13.634	13.634	.260	.005	.612
error	50	2623.001	52.460			
Within groups						
time of measurement	1	.955	.955	.177	.004	.676
time x groups	1	1.101	1.101	.204	.004	.654
error	50	270.337	5.407			

Significant at the p<0.05 level.

### Mean scores of victim's age

No significant main effect on time, *F*(1,50) = .001, *p* = .978, *η*^*2*^ *=* .000, and group, *F*(1,50) = .388, *p* = .538, *η*^*2*^ *=* .011 were found. There was also no significant time x group interaction effect, *F*(1,50) = 1.571, *p* = .219, *η*^*2*^ *=* .044. The results are shown in Table 5.

**Table 5:** Repeated measure ANOVA on mean scores of victim's age.

Source of variability	*df*	*SS*	*MS*	*F*	*η²*	*p*
Between groups						
group	1	25.903	25.903	.388	.011	.538
error	34	2270.576	66.782			
Within groups						
time of measurement	1	.006	.006	.001	.000	.978
time x groups	1	11.183	11.183	1.571	.044	.219
error	34	242.003	7.118			

Significant at the p<0.05 level.

### Polygraph test results

The polygraph test resulted in 24 participants falling under "No Significant Responses." and 2 participants under "Significant Response."

## Discussion

The purpose of the present study was to evaluate the extent to which the polygraph test would increase the number of disclosure on the sexual history in men who had committed sexual offenses. We examined within and between-group (polygraph vs. self-report) variabilities in the number of reports on sexual history.

The results showed that the SHDE interview with polygraph was more effective in collecting sexual history than a self-report. Although the increase was not dramatic, the13 out of 26 in the polygraph group (50%) reported more paraphilic interest behaviors. In particular, the number of reports on voyeurism and the searching target for sexual crimes increased. Since these behaviors are known to be closely associated with sexual crimes [30]; therefore, obtaining relevant information is critical for predicting and preventing recidivism.

The findings were consistent with the earlier researches [14, 22, 23, 31–34], indicating that the polygraph is a useful instrument for obtaining highly sensitive information that sex offenders did not want to disclose. Based on these results, the use of a polygraph test enables the SHDE interview to collect more information regarding the past sexual behavior and interest of offenders than self-report. Moreover, most participants relatively responded honestly on the previous sexual activities according to the result of the polygraph test. The findings from this study suggest that using a polygraph test would allow the investigator to conduct a more in-depth exploration of sexual activities that sex offenders previously engaged in but would not have disclosed before.

The reason the polygraph group disclosed more regarding their sexual histories is uncertain. It may be that they trusted the ability of the polygraph; therefore, they were more likely to disclose their past sexual activities. It may be explained by the theory of the Bogus pipeline [35], a method to reveal an examinee’s accurate responses without actually operating the machine. According to this study, just putting on the polygraph sensors leads people to show more truth. However, recent research on the Bogus pipeline investigated by Elliott, Egan, and Grubin [36] reported whether the level of polygraph accuracy was 100% or 75%, bogus lie detector group reported significantly more on cheating behavior than the control group. Thus, the bogus pipeline theory might not be fully explaining these results. It should be investigated further.

Nevertheless, in this study, regardless of the reason for increased disclosure, the polygraph verified the disclosed information. Therefore, the expectation of being detected by the polygraph helped offenders respond more openly and honestly. It will lead to collect valuable information on sex offender.

However, the offender's age at the early onset of criminal behaviors and the victim's age was not consistent with previous studies [21–23]. A possible explanation is that the participants in this study were not diagnosed as pedophiles. They were mostly accused of sexual assaults on the adult or mixed (adult and child) victims. Another possible explanation has to do with the traditional Confucian norms in Korean society. Although the internet and media changed the attitude on sexual behavior and engagement among young people last decades, premarital sex is still viewed negatively in the Korean culture. Thus, it assumed that the onset of the first crime could not have been younger due to the suppression of their sexual urges until adulthood. Thereby, the results did not show a significant difference in the victim and offender’s age at the early onset of criminal behaviors.

Discovering information on sexual behaviors and interests of sex offenders by utilizing the polygraph examination will help probation officers and sex offender treatment providers create specific management/treatment programs. Moreover, this will allow predicting offenders' future re-offending patterns [37]. Understanding the crime patterns of sex offenders through the acquired information helps organize a probation program. Despite the advantages of the polygraph test, it is not currently being utilized in sex offender management and treatment in South Korea. Therefore, the use of a polygraph test should be further expanded.

This study has several limitations. The generalization of results is difficult as the sample size of the participants was too small. Therefore, a large number of participants from different backgrounds must be included in future studies. In this study, even though the number of participants was small, most of them were recidivists who committed more than one sex crime. The use of the SHDE interview with a polygraph test is intended for deterring the re-offending of sex offenders. Thus, since most of the participants in this study were recidivists who committed more than one sex crime, it was appropriate.

Another limitation is that the information on participants’ experience on the polygraph was not included in this study. Rovner, Raskin, and Kircher [38] showed that experience on the polygraph might increase the number of false-negative responses due to the practice effect. However, some researchers reported that the polygraph's accuracy was not affected by practices [39]. Furthermore, the individual belief in the polygraph test leads to genuine and truthful responses [35]. Although the present study was unable to confirm the previous experience of participants’ polygraph use, it was able to identify that all of them had the first time to take the SHDE interview with a polygraph test.

Third, the available information was highly limited because it was an analysis of data collected by other agencies. Therefore, future investigations are necessary to collect varied information on participants to validate the conclusion.

Lastly, the environmental factors facing the two groups of participants were slightly different. In the case of the polygraph test group, the participants stayed for a month to assess for mental health investigation by court order. In contrast, the comparison group was imprisoned for treatment after the trial. In other words, the comparison group participants were fearless revealing their past behavior because they were already found guilty of sexual assaults by the court. However, participants in the polygraph group are afraid of disclosing new information because the trial is still underway. Nevertheless, the study presented that more participants from the polygraph group revealed a higher number of past sexual history than the comparison group. Consequently, the study confirms that the polygraph test's use is beneficial in disclosing more unrevealed information from participants.

Despite the limitations, the present study has significant implications. The study investigated the effectiveness of polygraph on the disclosure of sexual history among recidivists. Although the polygraph test is based on the participants’ self-reports, it helps determine the truthfulness of the information the offender provided. Furthermore, it can be obtained more numbers of information by enhancing participants’ self-openness. Based on these results, the polygraph test is proven it as a useful tool to disclose more information from sex offenders. Information obtained by the polygraph test will eventually help probation officer and sex offender treatment providers to plan the effective recidivism prevention programs.

Finally, the study's results partially supported the efficacy of PCSOT by confirming the effectiveness of the SHDE interview with the polygraph. As PCSOT is currently implemented in the United States and the United Kingdom, it should also be highly considered in South Korea to effectively control sex offenders' recidivism.

1

## Supporting information

S1 Appendix List of types of behavior discussed in sexual history disclosure examination. (DOCX)

S1 File Sexual history disclosure examination data set. (XLSX)
