# Correction: Improving the quality of sexual history disclosure on sex offenders: Emphasis on a polygraph examination

**DOI:** 10.1371/journal.pone.0256993

**Published:** 2021-08-30

**Authors:** Sue Hyun Jung, Min Jin Jin, Jang-Kyu Lee, Hee-Song Kim, Hyung-Ki Ji, Ki-Pyoung Kim, Myoung-Ho Hyun, Hyeon-Gi Hong

There are errors in the author affiliations. The correct affiliations are as follows:

Sue Hyun Jung^1,2^, Min Jin Jin^4^, Jang-Kyu Lee^3^, Hee-Song Kim^1^, Hyung-Ki Ji^1^, Ki-Pyoung Kim^1^, Myoung-Ho Hyun^2^, Hyeon-Gi Hong^1,2^

**1** Division of Forensic Psychology, National Forensic Service, Wonju-si, Gangwon-do, Republic of Korea, **2** Department of Psychology, Chung-Ang University, Seoul, Republic of Korea, **3** Institute of Forensic Psychiatry Ministry of Justice, Gongju-si, Chungcheongnam-do, Republic of Korea, **4** Institute of General Education, Kongju National University, Gongju, Republic of Korea.
