# Supplementary material for: Improving the quality of sexual history disclosure on sex offenders: Emphasis on a polygraph examination
Source: PLoS One. 2020 Sep 17;15(9):e0239046. doi: 10.1371/journal.pone.0239046 (PMC7498054; doi:10.1371/journal.pone.0239046)
Supplement: S1 Appendix — (DOCX) [file pone.0239046.s001.docx]

Appendix 1: List of Types of Behavior Discussed in SHDE

| 1. Sexual play with children (playing doctor or show me games) |
| --- |
| 1. Masturbation, How frequent? |
| 1. Masturbation in public (outdoors, restrooms, in vehicle) |
| 1. Masturbation to pornographic material |
| 1. Masturbation with under clothing/garments (stealing of clothes for masturbation) |
| 1. Voyeurism (peeking/watching for sexual purpose) |
| 1. Exhibitionism (exposing your sexual anatomy to others) |
| 1. Incest (any sexual activity with a family member) |
| 1. Homosexual behavior (any sexual activity with same sex, childhood or adult) |
| 1. Obscene phone calls, including prank or nuisance call |
| 1. Frottage (rubbing up against or touching others for sexual purposes) |
| 1. Molestation (any sexual contact with minors as adult) |
| 1. Setting fires (for sexual purposes) |
| 1. Prostitution (paying for sex with women) |
| 1. Stalking (willfully and repeatedly following someone) |
| 1. Transsexualism (thoughts or interest in wanting to be opposite sex) |
| 1. Transvestitism (activities involving dressing in opposite sex) |
| 1. Sadism (deriving sexual pleasure from another’s pain or humiliation) |
| 1. Mascohism (deriving sexual pleasure from receiving pain) |
| 1. Fetishism (sexual arousal from objects, -- underwear, feet, shoes, vibrators) |
| 1. Placing objects into anus for sexual arousal |
| 1. Urolagnia (use of urine for sexual excitement—golden showers) |
| 1. Coprophilia (use of feces for sexual excitement—brown showers) |
| 1. Cruising (driving, walking searching for a sexual target) |
| 1. Arousal to odors (any odor associated with sexual arousal) |
| 1. Animal sex or cruelty to animals |
| 1. Necrophilia (contact with dead animals or people) |
| 1. Sexual victimization (if you have been sexually abused) |
| 1. Contributed to the delinquency of minors—use of alcohol, drugs, shelter, porno |
| 1. Visiting areas where children frequent in order to have sexual contacts/fantasies |
| 1. Threesomes |
| 1. Visiting Nude Bars |
| 1. Sexual contact with fruit/vegetable |
| 1. Other |
| 1. Adultery |
| 1. Hurt anyone during a sexual experience |
| 1. Sexual contact with minors (under 13) when you were under 19 |
| 1. Sexual contact with minors (13 to 18) |
| 1. Forced sexual intercourse with patners |
| 1. Masturbation with celebrities’ picture or movie |
| 1. Pressure partner to drink alcohol for sexual purpose |
| 1. Sexual intercourse in public area (In the car, outside of mall) |
| 1. Text with minors or opposite sex for sexual purpose |
| 1. Sexual contact with Karaoke helpers |
| 1. Forced partner to mimic pornography |
